# Genetic Evaluation, Familial Screening and Exercise

**DOI:** 10.5935/abc.20170015

**Published:** 2017-03

**Authors:** Ricardo Stein, Juan Pablo Trujillo, Anderson Donelli da Silveira, Arsonval Lamounier Júnior, Lorenzo Monserrat Iglesias

**Affiliations:** 1 Programa de Pós-Graduação em Cardiologia e Ciências Cardiovasculares da Faculdade de Medicina da Universidade Federal do Rio Grande do Sul; Porto Alegre, RS - Brasil; 2 Grupo de Pesquisa em Cardiologia do Exercício do Hospital de Clínicas de Porto Alegre (CardioEx); Porto Alegre, RS - Brasil; 3 Serviço de Fisiatria e Reabilitação do Hospital de Clínicas de Porto Alegre - Universidade Federal do Rio Grande do Sul; Porto Alegre, RS - Brasil; 4 Vitta Centro de Bem Estar Físico, Porto Alegre, RS - Brasil; 5 Health in Code, La Coruña - Spain; 6 Grupo de Investigación Cardiovascular de La Universidad de La Coruña, La Coruña - Spain

**Keywords:** Genetics, Genotype, Exercise, Heredity, Sports Medicine, Death, Sudden, Cardiac

## Introduction

Regular physical activity practice benefits individuals of all ages, sexes and ethnicities.^1^ If on one hand the practice of moderate exercise is considered a healthy activity that favors the cardiovascular system, on the other, high-intensity exercise for a long period can increase the risk for sudden death (SD).^2^ Even considering the huge number of individuals exercising daily, SD in that context is rare. However, the prevention of SD can be difficult and it has significant repercussion, mainly among young practitioners of leisure exercise or athletes. From the epidemiological viewpoint, cardiac SD affects 200,000 to 400,000 individuals in the United States of America (USA) annually.^3^ In the sports scenario, around 200 athletes per year are estimated to have a fatal event.^4^ In Spain, the national registry of SD in athletes reported 180 cases from 1995 to 2007, suggesting an incidence of 15 to 20 cases per year.^5^

For athletes, preparticipation evaluation (PPE) is indicated, and can be effective in preventing cardiac SD in that context.^6^ However, that type of screening has great variability between different countries and entities that perform it. In the sports context, genetic evaluation is performed only in specific cases. 

This review describes basic aspects of genetic evaluation, as well as the indications for molecular analysis and their correct clinical interpretation, for practitioners of recreational exercise, amateur sportsmen and high-performance athletes.

### Sudden death of athletes: What diseases can be involved?

One of the major preoccupations in different sports modalities is to establish the risk of SD for each individual and if that modality can increase that risk. There might be a relationship between the sport modality and the cause of SD, which should be taken into consideration on the occasion of screening and prevention. Recent data estimate that among young North-American athletes (<35 years), the incidence of SD would be between 1 and 3 per 100,000 athletes.^7^ However, among athletes older than 35 years, that incidence can be greater, because the risk of SD due to ischemic heart disease increases progressively with age.

Few observational studies are available, most conducted in the USA, Italy, Spain and Denmark.^5,8,9^ Such studies agree on the identification of the different causes of SD among athletes aged less than or over 35 years. In the younger age groups, the most frequent causes are cardiomyopathies, channelopathies and coronary artery anomalies. However, in older age groups, the major cause is coronary artery disease (CAD), accounting for more than half of the cases of SD in that scenario.^10^ In Spain, according to the *Registro Nacional de Morte Súbita em Deportistas,*^5^ the major causes are: unidentified (27%), arrhythmogenic right ventricular cardiomyopathy (ARVC, 14%), hypertrophic cardiomyopathy (HCM, 12%), idiopathic left ventricular hypertrophy (8%), coronary artery anomalies (10%), aortic stenosis (6%) and myocarditis (4%). In Brazil, no epidemiological data on SD of athletes are available.

Thus, for young athletes, screening should focus on identifying inherited heart diseases, such as channelopathies and cardiomyopathies. For older individuals and the general population, however, that assessment should focus on diagnosing CAD.^11^

### Clinical Genetic Evaluation of the Sportsman

According to different expert opinions and consensus, genetic evaluation should not be routinely indicated for athletes. Considering that whether electrocardiography should be routinely indicated in PPE is still a matter of discussion, the performance of genetic evaluation should always be very well substantiated for the athlete.

Genetic evaluation is especially indicated on the following two occasions:

- positive family history of inherited heart disease (cardiomyopathies, channelopathies, aortopathies) or suspicion of that type of disease (syncope episodes, arrhythmias, cardiac arrest/SD). In such cases, the genetic evaluation should be first performed in the individual or in one of the affected relatives. Once detected the mutation causing the disease, the other family members should be assessed;
- when the athlete's phenotype strongly indicates the presence of an inherited disease (signs, symptoms and/or tests suggesting specific disease or compatibility with a disease).^12^

**Figure 1:**
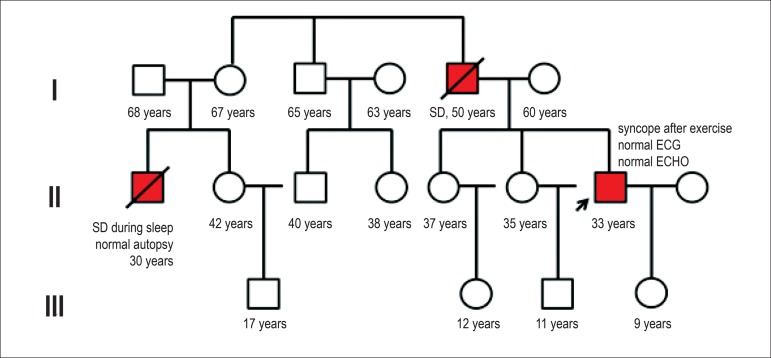
Pedigree chart of a family with clinical suspicion of channelopathy. Square: man; circle: woman; oblique bar: deceased; arrow: index-case; red circle or square: affected individual; SD: sudden death; ECHO: echocardiography.

Conducting a clinical genetic evaluation should always be the first step before performing a genetic test. That investigation should include the detailed assessment of family antecedents, as well as a complete physical examination. The family history should include the following: age at symptom onset; triggering activities; diagnosed disease; degree of kinship; and number of affected relatives. Building genealogical trees and family pedigree charts (Figure 1), representing family relationships, allows details on the ancestors; in addition, it is worth noting that the affected family side should always be the one investigated. If inherited heart disease is strongly suspected, but there is no suspected first-degree relative, the study should be extended to one more generation.

Thus, the clinician/cardiologist conducting the evaluations should be aware of the signs and symptoms of that group of diseases, and could even refer suspected cases to experts on family heart diseases and/or cardiovascular genetics. Delaying the diagnosis is not wanted, and physical exercise should be avoided in the period. It is worth emphasizing that the evaluation should not be restricted to the one individual diagnosed, but extended to his entire family.

### Genetic Cardiovascular Diseases

Inherited cardiovascular diseases, such as cardiomyopathies (hypertrophic, dilated, arrhythmogenic, restrictive and non-compacted), channelopathies [long QT syndrome (LQTS), Brugada syndrome (BrS) and catecholaminergic polymorphic ventricular tachycardia (CPVT)] and aortopathies (Marfan and Loeys-Dietz syndromes), are a group of entities of high clinical and genetic heterogeneity. Molecular studies performed in different populations have shown that each of those conditions associate with hundreds of different pathogenic mutations.^12^ There are mutations in different genes associated with the same phenotype. In some cases, the genes behave similarly or transcribe proteins that are part of the same structure or functional path (sarcomeric proteins, desmosomal junctions, ionic channels). In other cases, the presence of one single mutation can be enough for the disease development. It is worth noting that the clinical variability of the diseases can be explained by epigenetic factors and/or environment interaction. Finally, we emphasize that, the development of next generation sequencing (NGS), providing complete and parallel analysis of different genes, enables the identification of the causal genetic variant or variants of a disease in a faster and less expensive manner.^13^

#### Cardiomyopathies, Genetics and Sports

**Figure 2:**
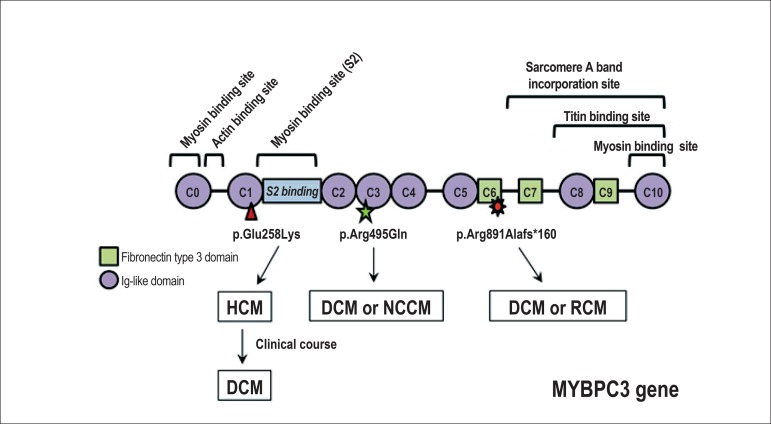
Clinical-molecular correlation of three pathogenic mutations in the MYBPC3 gene. One mutation can be associated with different cardiomyopathies. HCM: hypertrophic cardiomyopathy; DCM: dilated cardiomyopathy; NCCM: non-compacted cardiomyopathy; RCM: restrictive cardiomyopathy.

The European Society of Cardiology (ESC) defines cardiomyopathy as a myocardial disorder with structural and functional abnormalities, in the absence of CAD, hypertension, valvular disease and congenital heart disease sufficient to cause the observed myocardial abnormality. Some examples of cardiomyopathies are as follows: HCM; dilated cardiomyopathy (DCM); ARVC; restrictive cardiomyopathy; and non-compacted cardiomyopathy (NCCM). Those cardiomyopathies, except for ARVC, share sarcomeric gene mutations. Different pathogenic mutations in sarcomeric genes, such as MYH7 or MYBPC3, can be associated with several cardiomyopathies (Figure 2). In addition, one same mutation can be expressed with a different phenotype in different patients (even in the same family).

Non-sarcomeric genes can produce phenocopies. This is the case of the GLA gene, whose mutation causes Fabry's disease. That gene can be associated with HCM development (around 0.5-1% of the cases of HCM are explained by mutations in GLA gene).

The HCM is an autosomal dominant genetic disease. It is relatively common, with prevalence of 1:500 individuals in the general population. According to North-American data, that disease is the most frequent cause of SD in apparently healthy young individuals, especially athletes.^4^ In many cases, SD can occur during or right after exercise (approximately 40% of cases). Despite its catastrophic potential, however, the annual mortality rate in all patients with HCM is lower than 1%.^14^ A large number of mutations in different genes associated with HCM has been described. So far, hundreds of mutations in around 20 sarcomeric genes related to HCM have been identified (MYBPC3, MYH7, TNNC1, TNNT2 and TNNI3 are the most frequent genes). In addition, phenocopies of HCM derived from pathologies caused by mutations associated with the glycogen metabolism (PRKAG2, LAMP2) in storage diseases (GAA, GLA) and in mitochondrial genes have been reported.

The ARVC is characterized by ventricular myocardial tissue replacement with fibrous and adipose tissue, which has been associated with ventricular arrhythmias. The clinical diagnosis may be complicated, requiring extensive research for confirmation. From the epidemiological viewpoint, its prevalence ranges from 1:5,000 to 1:2,000 individuals.^15^ Most cases have an autosomal dominant pattern of inheritance. In some cases, however, as in Naxos disease, caused by pathogenic mutations in the plakoglobin (JUP) gene, that inheritance is autosomal recessive. ARVC has been associated with mutations in desmosomal (DSC2, DSG2, DSP, JUP, PKP2) and non-desmosomal (LMNA, CNLF, TMEM43 and PLN) genes. In that disease, regular and high-intensity exercise (competitive sports) has been associated with accelerated progression and worsening in animal and human models.^16,17^ Competitive sports increases by five times the risk for SD in adolescents and young adults with that disorder.^10^

#### Channelopathies, Genetics and Sports

Channelopathies are a group of diseases sharing some characteristics, such as genetic and clinical heterogeneity. Most of them are explained by changes in the genes that encode myocardial ionic channels. Some channelopathies are as follows: short-QT syndrome, LQTS, BrS and CPVT.

In LQTS, three most frequent subtypes were identified.^18^ In type 1 LQTS, patients can have cardiac events in adrenergic situations (in the sports context, swimming is a classic example). That is why the European and North-American guidelines recommend those individuals refrain from practicing water competitive sports. Mutations in the KCNQ1 gene (slow component of the delayed rectifier potassium channel, Kv7.1) has been associated with the development of that syndrome. In type 2 LQTS, patients can have cardiac events due to auditory stimuli (radio or telephone ring), and puerperal women can be more susceptible to auditory stimuli (newborn crying). Mutations in the KCNH2 gene (rapid component of the delayed rectifier potassium channel, Kv11.1) have been associated with the development of that syndrome. Less prevalent than the other two, type 3 LQTS has a parasympathetic substrate. In that subtype, patients can have cardiac events during resting periods or sleep. Mutations in the SCN5A gene (sodium channel gene, Nav1.5) have been associated with the development of that syndrome and with the BrS.

The arrhythmic events associated with the BrS usually occur during fever episodes, use of some medications, sleep or after exercise.^19^ Physical activity might have a pro-arrhythmic effect, associated with either hyperthermia or sympathetic withdrawal and/or increased vagal tonus in athletes after exertion. However, that association is still uncertain and the North-American guidelines do not recommend sports restrictions for those patients.

Finally, CPVT is associated with changes in intracellular calcium release from the sarcoplasmic reticulum. It is often expressed during the first decades of life, manifesting as syncope or SD associated with exercise and/or stressful situations. Therefore, international guidelines recommend strict sports restrictions in such cases.^20^ Patients with CPVT can have mutations in the RYR2 (major), CASQ2 and KCNJ2 genes.

#### 2.3. Inherited Aorta Diseases, Genetics and Sports

This group of diseases includes a set of inherited connective tissue disorders that predispose to aortic dilations and aneurysms (AA) and/or dissection (AD), with an increased risk for SD during physical activity. Some of those diseases are rare genetic syndromes (Marfan, Loeys-Dietz and vascular type Ehlers-Danlos) and no-syndromic presentations, such as familial thoracic aortic aneurysm disease.

When performing PPE in patients with aortopathies classified as syndromic, the variability or phenotypical overlapping of those diseases should be considered. Identification of some of the following clinical signs in an individual who would apparently have a normal phenotype can contribute to the differential diagnosis:^21^ Marfanoid *habitus*; kyphosis/scoliosis; changes in skin elasticity and/or joints; *ectopia lentis*; and craniofacial dysmorphism. In a case report, abdominal AA was detected in an elite athlete of North-American basketball after the late diagnosis of Marfan.^22^ In another case, a young weightlifter died suddenly from an AD, and upon autopsy was diagnosed as having "non-Marfan's fibrillinopathy". His echocardiogram was normal, but his mother had died at a young age, also from AD.^23^ Other authors^24^ have reported a family with three generations affected (Marfan). The diagnosis was established only after AD findings in a 30-year-old weightlifter. Both his father and brother had died on separate occasions following loss of consciousness after weight lifting.

Phenotypic overlapping can occur in those diseases, as seen with the Loeys-Dietz and Marfan's syndromes. Differentiating between them is important to establish the prognosis and regularity of the clinical-cardiological follow-up. In patients with Marfan's syndrome, it is essential to keep watch on the aortic diameter in relation to the body surface,^25^ arterial stiffness, and ventricular function.^26^ In patients with Loeys-Dietz syndrome, a systematic assessment of aneurysms in multiple arteries is necessary. Referring to an expert in medical genetics or to a specialized center helps in the management of those patients.^25^ The genetic exam helps to define borderline or dubious cases (which do not meet the criteria for the clinical diagnosis of that syndrome), in addition to aiding in the differential diagnosis of aortopathies.^27^

In athletes with Marfan's syndrome or other aortopathies, AD or aortic rupture can cause SD. The increase in aortic blood pressure and stress during exercise, in the presence of genetic predisposition, can accelerate aneurysm formation, serving as a trigger for AD/rupture of the aorta or of other arteries. A cohort of individuals admitted with AD to the emergency unit has evidenced that syndromic individuals are at higher risk for recurrence and death as compared to non-syndromic ones.^28^ Guidelines recommended that, in general, athletes with increased aortic diameter (> 40 mm in adults) only participate in low-intensity dynamic and static sports (class IA sports).^29^ A study has followed up a cohort of 732 individuals with Marfan's syndrome, all of them on pharmacological treatment, for 6 years. The risk for aortic events and SD remained low in those with aortic diameter between 35 and 49 mm. However, an aortic diameter of 50 mm has been described as the cut-off point for indicating prophylactic surgery.^30^ Finally, management based on the aortic diameter has been proposed, not only for Marfan's syndrome, but also for other aortopathies, such as familial thoracic aortic aneurysm disease and Ehlers-Danlos syndrome.^31^

### Usefulness and Limitations of Genetic Testing in Inherited Heart Diseases

Currently, with the emergence of NGS, diseases with high clinical and genetic heterogeneity can be studied faster and more accurately. This sequencing technology allows the construction of panels that capture the genes involved in each group of diseases (specific genetic panels for cardiomyopathies, channelopathies or aortopathies). In addition, enlarged panels directed to the study of SD with either structural or non-structural heart disease are useful in this context.^13^

In the presence of evident clinical findings that raise the suspicion of a particular disease, diagnostic genetic testing is usually more likely to confirm it (high pretest likelihood). Regarding the performance of genetic tests in primary cardiomyopathies, using a well-designed panel, mutations can be identified in up to 70% of the cases of HCM, for example. Other genes associated and the pretest likelihood of their identification in cardiomyopathies, channelopathies and aortopathies are shown in Tables 1, 2 and 3.

**Table 1:** Genes frequently associated with the development of different cardiomyopathies

Cardiomyopathy	Gene (symbol)	Pretest likelihood
HCM	MYBPC3, MYH7, TNNC1, TNNT2, TNNI3, TPM1, ACTC1, MYL2, MYL3, PRKAG2, LAMP2, GLA, GAA, TTR, PTPN11.	70%
DCM	TTN, ACTC1, BAG3, DES, DMD, DSP, FLNC, LMNA, MYBPC3, MYH7, PKP2, PLN, RBM20, TAZ, TNNC1, TNNT2, TNNI3, TPM1.	40 - 50%
ARVC	DSC2, DSG2, DSP, JUP, PKP2, LMNA, FLNC, TMEM43, PLN.	50 - 65%
NCCM	MYBPC3, MYH7, ACTC1, TAZ, LDB3.	40 - 50%

HCM: Hypertrophic cardiomyopathy; DCM: Dilated cardiomyopathy; ARVC: Arrhythmogenic right ventricular cardiomyopathy; NCCM: Non-compacted cardiomyopathy.

**Table 2:** Genes frequently associated with the development of different channelopathies

Channelopathy	Gene (symbol)	Pretest likelihood
LQTS	KCNQ1, KCNH2, SCN5A, KCNJ2, KCNE1, KCNE2, CACNA1C.	70%
SQTS	KCNH2, KCNQ1, KCNJ2	Unknown
BrS	SCN5A, SCN10A	30%
TVPC	RYR2, CASQ2, KCNJ2	50 - 60%

LQTS: Long QT syndrome; SQTS: Short QT syndrome; BrS: Brugada syndrome; CPVT: Catecholaminergic polymorphic ventricular tachycardia.

**Table 3:** Genes frequently associated with the development of different genetic aortopathies

Genetic aortopathies	Gene (symbol)	Pretest likelihood
Marfan’s syndrome	FBN1	~70 - 93%
Loeys-Dietz syndrome	TGFBR2, TGFBR1, SMAD3, TGFB2, TGFB.	~70 - 95%
Vascular-type Ehlers-Danlos syndrome	COL3A1	>95%
Familial thoracic aortic disease	ACTA2, TGFBR2, TGFBR1, MYH11, SMAD3, MYLK, FBN1	~17 - 20%

### Clinical Interpretation of the Results of Genetic Studies

Proper interpretation of the results of a genetic test is essential not only to establish the correct diagnosis, but also to properly guide athletes and their families. Therefore, careful assessment of the pathogenesis of a variant (Table 4)^32^ is a key aspect. All information available at major databases and publications should be taken into consideration, and that information should be analyzed by a skilled team, ensuring a reliable result.

**Table 4:** Clinical significance of the variant according to available information (Modified from *Standards and guidelines for the interpretation of sequence variants: a joint consensus recommendation of the American College of Medical Genetics and Genomics and the Association for Molecular Pathology*)^32^

Classification of the variant	Classification criteria	Clinical usefulness
Pathogenic	Not identified in the general population; variant widely described in the literature, with cosegregation demonstrated and strong evidence of genotype-phenotype association. Deleterious functional studies.	- Predictive clinical value. - Widely available clinical information. - Inclusion in familial screening is recommended. - Useful in PGD*.
Very likely pathogenic	Not identified in the general population; likely cosegregation of the variant in at least one family, truncating-type or in frame ins/del mutation in genes described with genotype-phenotype association that explains the patient’s disease. Deleterious functional study.	- Predictive clinical value. - Inclusion in familial screening is recommended. - Limitation in PGD (elucidation on expressivity and incomplete penetrance).
Likely pathogenic	Absent truncating-type or in frame ins/del mutation or identified in the general population with very low allele frequency (<0.01%); intronic variant that affects splicing. Genotype-phenotype association documented in at least two individuals.	- No predictive clinical value. - Allows cosegregation study in the family, which might aid in defining the pathogenesis.
Uncertain clinical significance	Variant with contradictory information on its pathogenesis, does not meet the criteria to be included in another category of the classification.	- No predictive clinical value. - Allows cosegregation study in the family, in the investigation context at the attending physician’s discretion.
Likely non pathogenic or benign	Allele frequency of the variant in control populations is higher than expected for the pathology. Absence of cosegregation. Missense variant in one gene, where only radical mutations are considered pathogenic. Benign functional study.	- No predictive clinical value. - Inclusion in familial screening not recommended.
Non pathogenic or benign	High frequency in the control population or previously described as benign. Absence of cosegregation. Benign functional study.	- Benign variant. - Should not be included in familial screening.

* PGD: Pre-implantation Genetic Diagnosis.

It is consensus that, in case of uncertainty about the pathogenesis of the mutation (uncertain clinical significance), it should be used for neither disease diagnosis nor familial screening (no clinical predictive value). In addition, some cases are of difficult solution, even after proper identification of the pathogenic variants. International guidelines disagree about athletes who are clinically healthy or not affected (negative phenotype), but carry the pathogenic variant (positive genotype). Considering the early diagnosis in athletes with positive genotype and negative phenotype, the North-American guidelines are much more liberal, and often do not disqualify those athletes for competitive sports. The ESC guideline, however, is much more restrictive.^33^

#### What to do if an athlete has a positive genetic test?

The presence of a genetic mutation in an athlete does not mean the athlete will develop the disease, but increases his susceptibility to develop it. Sometimes, not all carriers of a mutation develop the disease (incomplete penetrance). Some mutations require additional environmental (sports, hypertension) or genetic factors (presence of other mutations in the same or other genes). In the following situations an athlete can test positive for a genetic study:

- The athlete clearly has a familial heart disease (cardiomyopathy, channelopathy or aortopathy). The presence of a positive genetic test will confirm the diagnosis and help in screening the athlete's family.
- There is a previous diagnostic suspicion that the athlete has a family heart disease. A positive genetic test might help in establishing the definitive diagnosis and identifying whether the mutation is pathogenic, very likely pathogenic, or likely pathogenic.
- The athlete has no clinical manifestation of the disease, but an affected first- or second-degree relative. A positive genetic test in the family's index-case will confirm or discard that variant in the athlete.

#### What to do if an athlete has a negative genetic test?

Absence of a genetic variant does not rule out the disease in the athlete. In the following situations an athlete can test negative for a genetic study:

- The athlete clearly has a familial heart disease. In the presence of a negative genetic test, other genes that have not been described may be involved. In this case, the percentage of positivity (yield) of currently available genetic studies (in the presence of disease), which vary according to different pathologies (Tables 1, 2 and 3), should be considered.
- There is a previous diagnostic suspicion that the athlete has a family heart disease. A negative genetic test confirms the absence of disease in this individual, although a follow-up is recommended, at least annually, if there is a borderline change in previous diagnostic tests.
- The athlete has no clinical manifestation of the disease, but an affected first- or second-degree relative. A negative genetic test in the family's index-case will not allow proper familial screening. Thus, predisposition to develop the disease can be neither confirmed nor ruled out. In this case, follow-up is recommended, at least annually, especially if there is a borderline change in previous diagnostic tests.

## Conclusion

**Figure 3:**
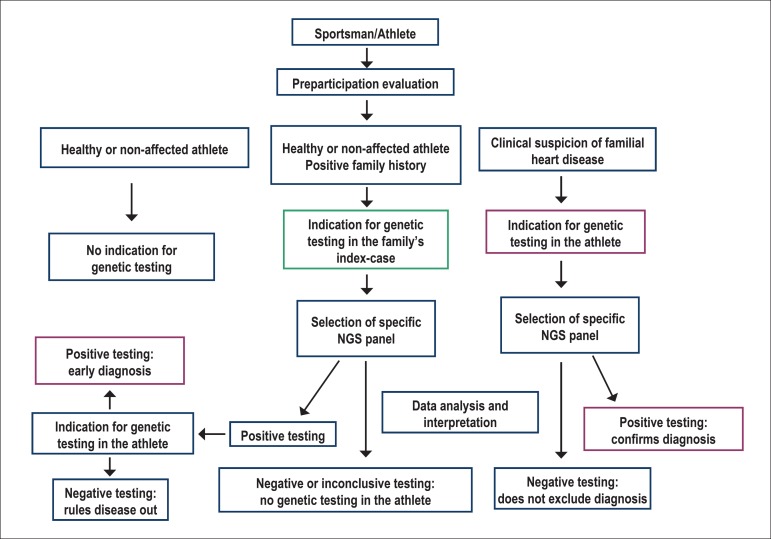
Possible clinical scenarios in the context of an athlete's genetic testing.

Genetic studies have become an instrument to help in the diagnostic confirmation of different inherited heart diseases. Physicians, including those of sports and exercise medicine, however, should know very clearly their indications and limitations in clinical practice. In PPE, complete history (individual and family) and a detailed physical exam, in addition to complementary tests, should always precede the application of genetic analysis (Figure 3). There is consensus that genetic testing, at least as a routine process, is not indicated in athletes. Its use is clearly indicated only in two particular cases: a) athletes with suspected or definite diagnosis of a familial disease; b) healthy or non-affected athletes, with a positive family history of an inherited disease, as part of the familial screening.

In the sports context, it is essential to consider that the correct interpretation of the genetic tests will reduce false-positive and false-negative results. This can prevent incorrect interpretation and recommendations, inappropriate disqualifications or unwanted events (SD, for example). We hope that, in the future, when the epidemiological and molecular aspects of these diseases are better known, a better genotype/phenotype correlation by use of genetic studies will be available. Therefore, it is necessary to create and foster multidisciplinary teams dedicated to information management and analysis, aimed at elaborating effective SD prevention programs for individuals who exercise in a recreational way, as well as for amateur and professional athletes.
